# Effects of simulated microgravity on biological features and virulence of the fungal pathogen *Cryptococcus neoformans*

**DOI:** 10.1128/aem.01435-25

**Published:** 2025-09-30

**Authors:** Tanaporn Phetruen, Salinthip Thongdechsri, Muthita Khongthongdam, Sittiporn Channumsin, Krai Meemon, Sittinan Chanarat

**Affiliations:** 1 Laboratory of Medical Molecular Mycology, Department of Biochemistry and Center for Excellence in Protein and Enzyme Technology, Faculty of Science, Mahidol University, Bangkok, Thailand; 2 Department of Anatomy, Faculty of Science, Mahidol University 546354, Bangkok, Thailand; 3 Space Technology Research Centre, Geo-Informatics and Space Technology Development Agency (GISTDA), Chonburi, Thailand; Centers for Disease Control and Prevention, Atlanta, Georgia, USA

**Keywords:** *Cryptococcus neoformans*, simulated microgravity, stress tolerance, virulence factors, pathogenicity, *Caenorhabditis elegans*

## Abstract

*Cryptococcus neoformans* is a fungal pathogen that causes cryptococcal meningitis, mainly in immunocompromised individuals, such as those with HIV. Its recent detection on the International Space Station raises concerns about potential health risks in space, where immune systems may be compromised. However, its behavior in space-like conditions remains unclear. In this study, we examined the effects of simulated microgravity on *C. neoformans*. We found that the condition enhanced the fungus’s resistance to membrane and osmotic stress and increased key virulence factors, including capsule formation, melanin production, and urease activity. Using *Caenorhabditis elegans* as a host model, infections under simulated microgravity were more pathogenic. These findings highlight the potential for increased fungal virulence in space and underscore the need to understand microbial risks for astronaut health and safety in long-term space missions.

**IMPORTANCE:**

Fungi have long been recognized for their remarkable ability to adapt to a wide range of environmental conditions, including extreme environments, such as space habitats. Understanding how fungal organisms, especially pathogenic fungi, adapt to these harsh conditions is crucial for gaining insight into their tolerance mechanisms and the potential emergence of virulence. Our research demonstrates that the pathogenic fungus *Cryptococcus neoformans* not only survives in space-like conditions but also exhibits increased stress tolerance, enhanced expression of key virulence factors, and elevated pathogenicity in animal models. These findings carry significant practical implications because concerns about fungal contamination in space or other extreme environments may be heightened by the potential for fungi to develop increased virulence through natural adaptation.

## INTRODUCTION

*Cryptococcus neoformans* is an encapsulated yeast pathogen that causes cryptococcosis, which occurs primarily in the lung and central nervous system, known as cryptococcal meningitis (1, 2). The global burden of cryptococcal meningitis is approximately 250,000 cases, with mortality rates of 100% if infections remain untreated, resulting in about 200,000 deaths annually (3). Despite these alarming statistics, both research uncovering the infection mechanism of *C. neoformans*, including the traversal to the blood-brain barrier, and the treatment options for cryptococcosis remain limited, with only three major classes of drugs approved for clinical use. The individuals most susceptible to *C. neoformans* infection are severely immunocompromised. Therefore, the importance of *C. neoformans* as an opportunistic pathogenic fungus has considerably increased over the last two decades, owing to the intensive chemotherapy of cancer patients, the use of immunosuppressive drugs in organ transplant recipients, and the spread of AIDS epidemics (3–5).

Beyond underlying immunodeficiency caused by diseases, the human body can become immunocompromised under extreme conditions. Specifically, living in spaceflight or on space stations can induce this stage (6). These include ionizing radiation and alterations in gravitational fields, which can profoundly impact the immune system (7–12). Research has shown that ionizing radiation and microgravity reduce the number of important immune cells, such as dendritic cells and natural killer cells (13), impair macrophage differentiation, and inhibit antigen presentation (14). Furthermore, prolonged exposure to microgravity leads to changes in immune response signaling pathways that subsequently affect T cells' number and activation processes, potentially enhancing immune dysregulation (15, 16). These alterations compromise the ability of the immune system to defend against pathogens. Thus, the immunocompromised state induced by space environments potentially escalates the risk and frequency of microbial infections, particularly opportunistic fungal infections, posing substantial challenges to human health during space missions.

Studies have confirmed the presence of fungi on environmental surfaces, including those within the International Space Station (ISS), using both morphological analysis and molecular techniques like 18S- and ITS1-rDNA sequencing (17–19). Among those fungal species, medically relevant *Cryptococcus* species have been identified (20). However, research specifically focused on *Cryptococcus* within space habitats and its response to space environments is currently limited. The only study conducted in *C. neoformans* revealed that melanized cells exhibited 50% higher viability compared to non-melanized cells after 29 days aboard the ISS, underscoring the protective properties of melanin against space-induced stressors (21). Nevertheless, the lack of research in this area emphasizes a notable gap in our understanding of how *C. neoformans* behaves and potentially adapts to space conditions.

To improve human biological safety during space missions, it is crucial to understand how *C. neoformans* behaves in space environments. In this study, we explore the impact of simulated microgravity as a single simulated environmental factor on the biology and virulence of *C. neoformans*. This approach allows us to observe the fungal growth phenotype and stress tolerance, which shows that membrane and osmotic stress tolerance are enhanced under simulated microgravity conditions. Furthermore, we have observed that several virulence factors of *C. neoformans* increase when exposed to simulated microgravity, which also affects fungal pathogenicity *in vivo* using *Caenorhabditis elegans* as a model. Taken together, this research provides insights into how microgravity-like conditions affect *C. neoformans*, aiming to deepen our understanding and develop strategies for managing this organism both in space and on Earth.

## RESULTS

### Simulated microgravity condition does not affect the growth phenotype of *C. neoformans*

**Fig 1:**
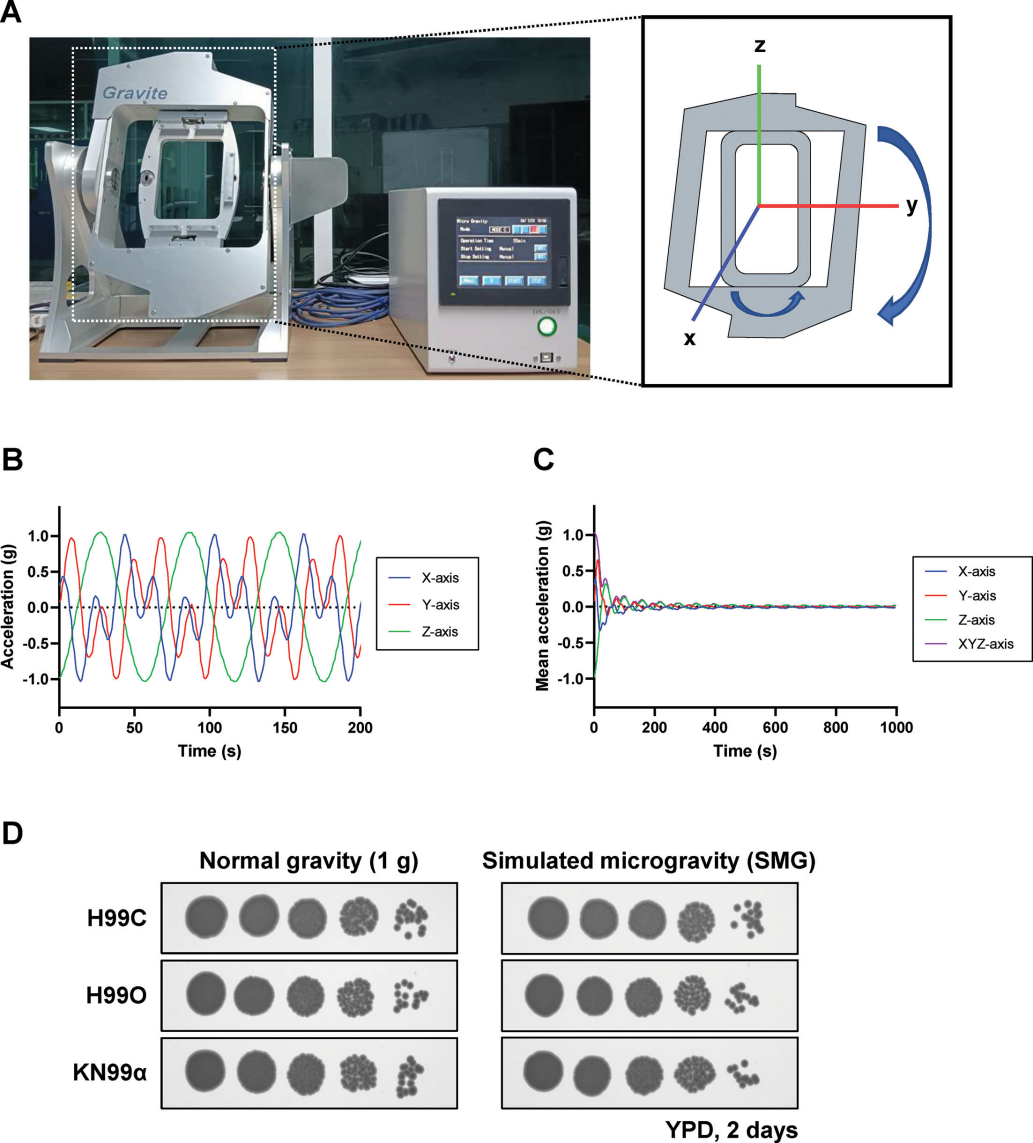
Simulated microgravity conditions do not affect the growth of *C. neoformans*. (**A**) Left, the 3D clinostat machine was used to simulate the microgravity conditions in this study. Right, the diagram represents the plates continuously rotating both axes in the same direction. (**B**) Acceleration graph over time for each axis. During rotation of the clinostat, the accelerations along the x, y, and z axes showed continuous variation, ranging from −1 to +1 g. (**C**) Mean acceleration graph over time for each axis and combination of three axes. The average acceleration decreased over time as acceleration accumulated in all directions and reached stability at 0.01 g after 600 s of machine operation. (**D**) Growth of *C. neoformans* strains H99C, H99O, and KN99α as representatives of low, intermediate, and high virulence strains, respectively, under normal gravity (1 g) and simulated microgravity (SMG) conditions. Ten-fold serial dilutions of cells were spotted on YPD plates and incubated at 25°C for 2 days.

The 3D clinostat (Gravite, Space Bio Laboratory) was developed to create a simulated microgravity environment on Earth to mimic the conditions in space flights and the International Space Station (ISS) (22). This machine provides the simulated micro-weight or microgravity conditions by continuously rotating both axes at a constant speed (Fig. 1A). It will allow unidirectional gravitational force to diverge in various directions and minimize the cumulative gravity vector at the center of the device, thus creating a simulated microgravity environment (23). The 3D acceleration sensor in the machine records the change along the x, y, and z axes. It monitors real-time acceleration value, in which an average G value of all axes is initially high and subsequently decreases and converges to zero before reaching stability at 0.01 g within 600 s or 10 min after machine operation (Fig. 1B and C). This data reveals that the 3D clinostat is capable of simulating and maintaining a constant simulated microgravity environment for our study.

Three strains of *C. neoformans,* including H99C, H99O, and KN99α, were used as representative models of low, intermediate, and high virulence, respectively. Each strain was serially diluted and spotted onto the YPD agar before incubating under simulated microgravity (SMG) conditions for 2 days. The culture incubated on normal gravity (1 g) was used as a control. The growth phenotype and colony morphology revealed similar manners in both SMG and control conditions (Fig. 1D). We expanded the observation to *C. neoformans* cultured in broth media. It is important to note that the media needs to fill the top of the T25 culture flasks with no air bubbles to minimize the possibility of turbulence and shear forces during culture rotation (13). However, *C. neoformans* is an obligate aerobic fungus that requires atmospheric levels of oxygen, or approximately 21% for optimal growth (24). Full culture media in the culture flask reduces the circulating oxygen, resulting in decreases in the growth rate and leading to the abnormality of cells, i.e., cell size reduction and irregular cell shape in both normal gravity and SMG conditions (data not shown). Therefore, we decided to use solid media in other experiments of this work.

### Simulated microgravity increases membrane and osmotic stress tolerance in *C. neoformans*

Previous research has reported the effects of space exposure on ISS, radiation, and microgravity on the stress response of different microbial species, including fungi (25–27). However, the response to abiotic stresses of *C. neoformans* has never been described. In this study, we investigate the effects of SMG on multiple stress tolerances, including membrane, osmotic, oxidative, and pH stresses in *C. neoformans*. Different degrees of stress were set based on the maximum and minimum levels that cells can tolerate in normal growth conditions.

**Fig 2:**
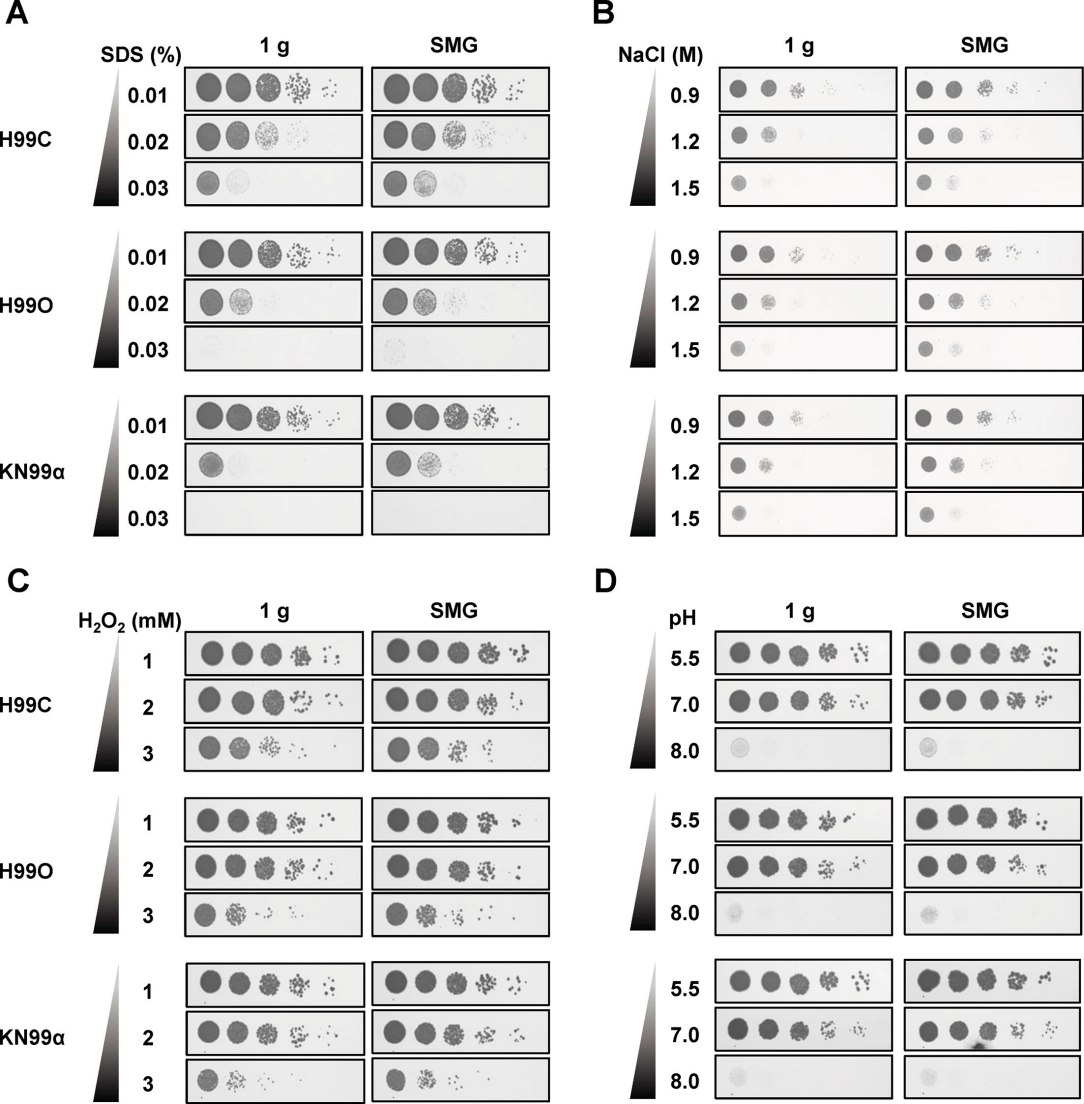
Stress tolerance of *C. neoformans* slightly changes under simulated microgravity conditions. *C. neoformans* strains H99C, H99O, and KN99α were 10-fold serially diluted (1‒10^4^ dilutions) and spotted on YPD medium containing the indicated stress-inducing agents: (**A**) SDS for membrane stress, (**B**) NaCl for osmotic stress, (**C**) H_2_O_2_ for oxidative stress, and (**D**) various pH levels achieved using NaOH for pH stress. Cells were incubated under simulated microgravity and normal gravity conditions for 2 days at 25°C and then photographed. Figures are representative of duplicate experiments. 1 g, normal gravity; SMG, simulated microgravity conditions.

The tolerance to membrane stress of *C. neoformans* strains H99C and H99O under normal gravity conditions was approximately 0.02% SDS, consistent with previous findings (28) (Fig. 2A). However, spot testing revealed that colonies grew better at 0.02% SDS under SMG than under normal gravity conditions (Fig. 2A and Fig. S1A). Similarly, strain KN99α, which tolerates up to 0.03% SDS, showed increased colony numbers under SMG in all concentrations of SDS. These results indicate that SMG enhances membrane stress tolerance across all three tested strains of *C. neoformans*.

We applied NaCl at final concentrations ranging from 0.9 to 1.5 M to assess osmotic stress tolerance (Fig. 2B). Three strains of *C. neoformans* were resistant to 1.5 M NaCl (28, 29), and enhanced growth was observed under SMG conditions, similar to the findings in membrane stress tolerance (Fig. 2B and Fig. S1B). We then imply that SMG conditions enhance osmotic stress tolerance in *C. neoformans*.

Next, we induced oxidative stress using hydrogen peroxide (H_2_O_2_) at the final concentrations ranging from 1 to 3 mM (Fig. 2C). All strains of *C. neoformans* examined in this study exhibited growth defects with increasing H_2_O_2_ concentration, yet they could tolerate up to 3 mM of H_2_O_2_ (30). While no apparent difference was observed between cultures grown under SMG and normal gravity conditions, slightly higher cell density and size were noticeable under SMG (Fig. 2C; Fig. S1C).

Similar results were observed with pH stress, where we adjusted the pH of YPD media by adding 2 M NaOH, starting at a pH of 5.5 for the freshly prepared media. We found no observable difference in pH stress tolerance between SMG and normal gravity conditions (Fig. 2D). Although there was a slight increase in colony growth under microgravity simulated by clinorotation, we observed that this effect was not as strong as those seen in membrane and osmotic stress tolerance (Fig. 2D; Fig. S1D).

From the above findings, we conclude that SMG conditions enhance membrane and osmotic stress tolerance in *C. neoformans*, while demonstrating minimal effects on oxidative stress and nearly no effect on pH stress tolerance.

### Simulated microgravity conditions enhance susceptibility to certain antifungal agent

Intrigued by the above findings, we speculated that the effect of SMG on membrane and osmotic stress tolerance may be linked to the susceptibility to antifungal agents, particularly the antifungal agents targeting fungal cell walls and cell membranes. We then used three major antifungals, amphotericin B (AMB), fluconazole (FLZ), and 5-fluorocytosine (5FC), the first-line treatments for cryptococcal meningitis (31, 32), to test the antifungal susceptibility under SMG and normal gravity conditions.

**Fig 3:**
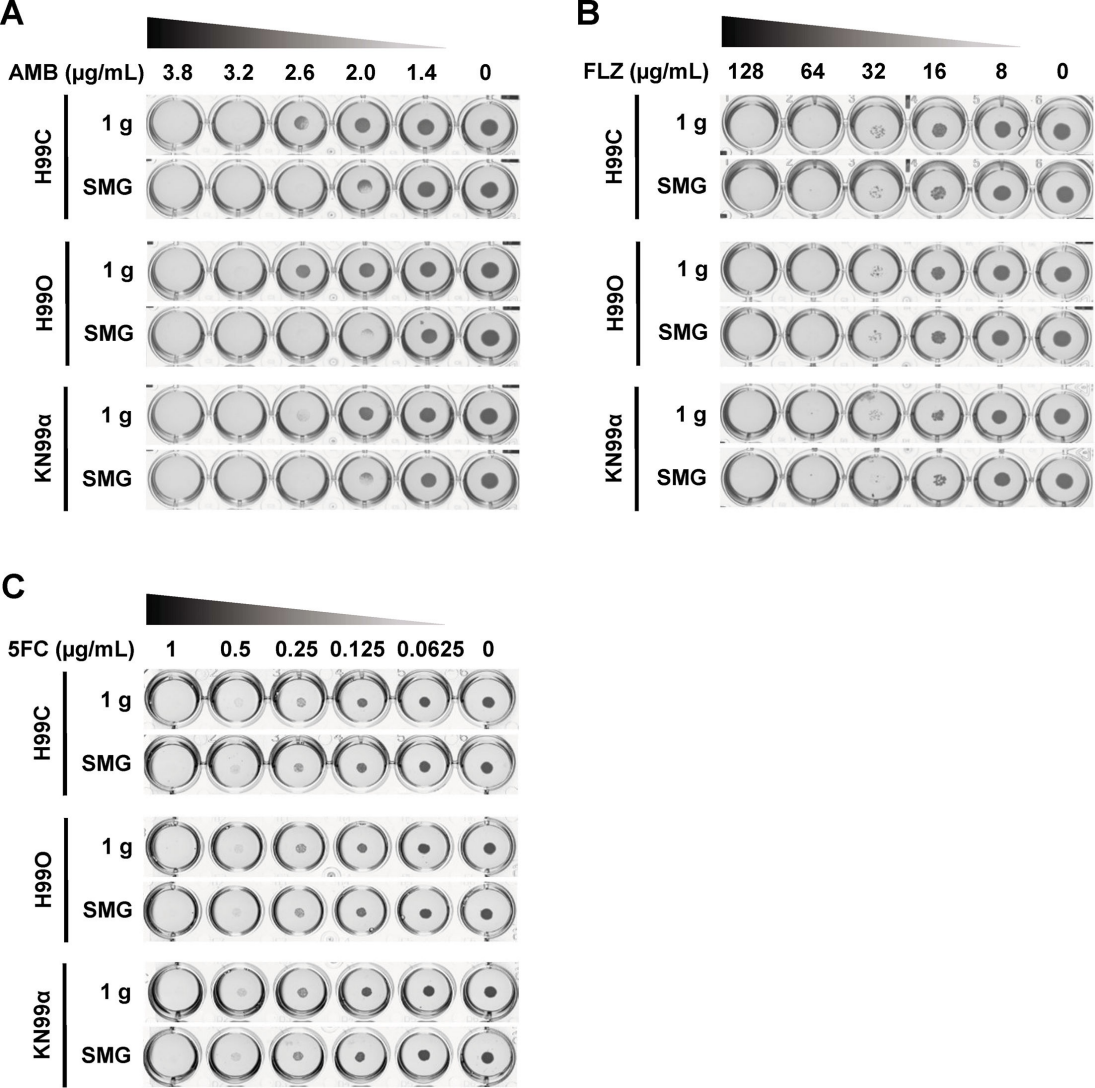
Simulated microgravity condition increases antifungal susceptibility in *C. neoformans*. Each strain was diluted to 0.5 on the McFarland scale and spotted on medium supplemented with the indicated concentration of (**A**) amphotericin B (AMB), (**B**) fluconazole (FLZ), and (**C**) 5-fluorocytosine (5FC). Cells were incubated under simulated microgravity and normal gravity conditions for 2 days at 25°C and then photographed. Figures were the representatives of duplicate data. 1 g, normal gravity; SMG, simulated microgravity conditions.

Amphotericin B belongs to the polyene class of antifungals. It binds directly to ergosterol in the fungal cell membrane, leading to pore formation, ion leakage, and eventual fungal cell death (33, 34). In our study, we prepared YPD agar in a 24-well plate supplemented with varying concentrations of AMB ranging from 0.125 to 2 µg/mL. We then spotted diluted *C. neoformans* cultures and incubated them for 2 days. The minimal inhibitory concentration (MIC) of all three strains under normal gravity and SMG conditions seemed to be ≥2 µg/mL (Fig. S2). Interestingly, at an AMB concentration of 2 µg/mL, fungal growth was lower under SMG compared with the control. We then tried to distinguish this result by using the AMB ranging from 1.4 to 3.8 µg/mL instead. The MIC of AMB under normal gravity conditions was 2.6 µg/mL (Fig. 3A; Fig. S3A). However, under SMG conditions, the MIC was reduced to 2 µg/mL. These results were surprising, indicating that simulated microgravity conditions increase sensitivity to AMB in *C. neoformans* while paradoxically enhancing tolerance to membrane stress, as shown in the previous results. We then hypothesize about the amount of membrane ergosterol, the major target of AMB, that may be affected by the SMG condition. Therefore, we extracted and measured the percentage of ergosterol per total protein weight. We found that the culture growing under SMG has a significantly higher amount of ergosterol compared with the control (Fig. S4). This potentially contributes to higher susceptibility to AMB of *C. neoformans* under SMG conditions.

Fluconazole, a member of the azole group, functions by interacting with 14-demethylase, a fungal cytochrome P-450 enzyme. This interaction disrupts the conversion of lanosterol to ergosterol, thereby compromising fungal cell membranes (35, 36). We applied FLZ at final concentrations ranging from 8 to 128 µg/mL. The MICs of all strains were found to be 32 µg/mL under both conditions (Fig. 3B; Fig. S3B), indicating that SMG does not change the susceptibility to fluconazole compared with the normal gravity conditions.

5-Fluorocytosine or flucytosine is an antifungal medication in the pyrimidine class. It is extensively incorporated into fungal RNA as 5-fluorouridine triphosphate and inhibits the biosynthesis of DNA, RNA, and proteins (37). We supplemented 5FC at concentrations ranging from 0.0625 to 1 µg/mL in RPMI-1640 medium. Similar to fluconazole, the MICs of all strains were 0.5 µg/mL under both SMG and normal gravity conditions (Fig. 3C; Fig. S3C), suggesting that SMG does not affect the susceptibility to flucytosine.

From these findings, we concluded that SMG neither affects sensitivity to FLZ nor 5FC of *C. neoformans*. However, it enhanced sensitivity to AMB in this fungal pathogen. This suggests that microgravity simulated by clinorotation may influence the integrity or components of fungal cell membranes, thereby impacting its response to antifungal agents like AMB.

### Virulence factors of *C. neoformans* increase under simulated microgravity conditions

Several virulence factors have been identified that contribute to increasing the pathogenicity of *C. neoformans*. These features include capsule formation, capsule polymorphism, melanin production, and the capability to secrete urease. Each of these factors plays a crucial role in the fungus’s ability to survive and cause disease in its host (38–40).

**Fig 4:**
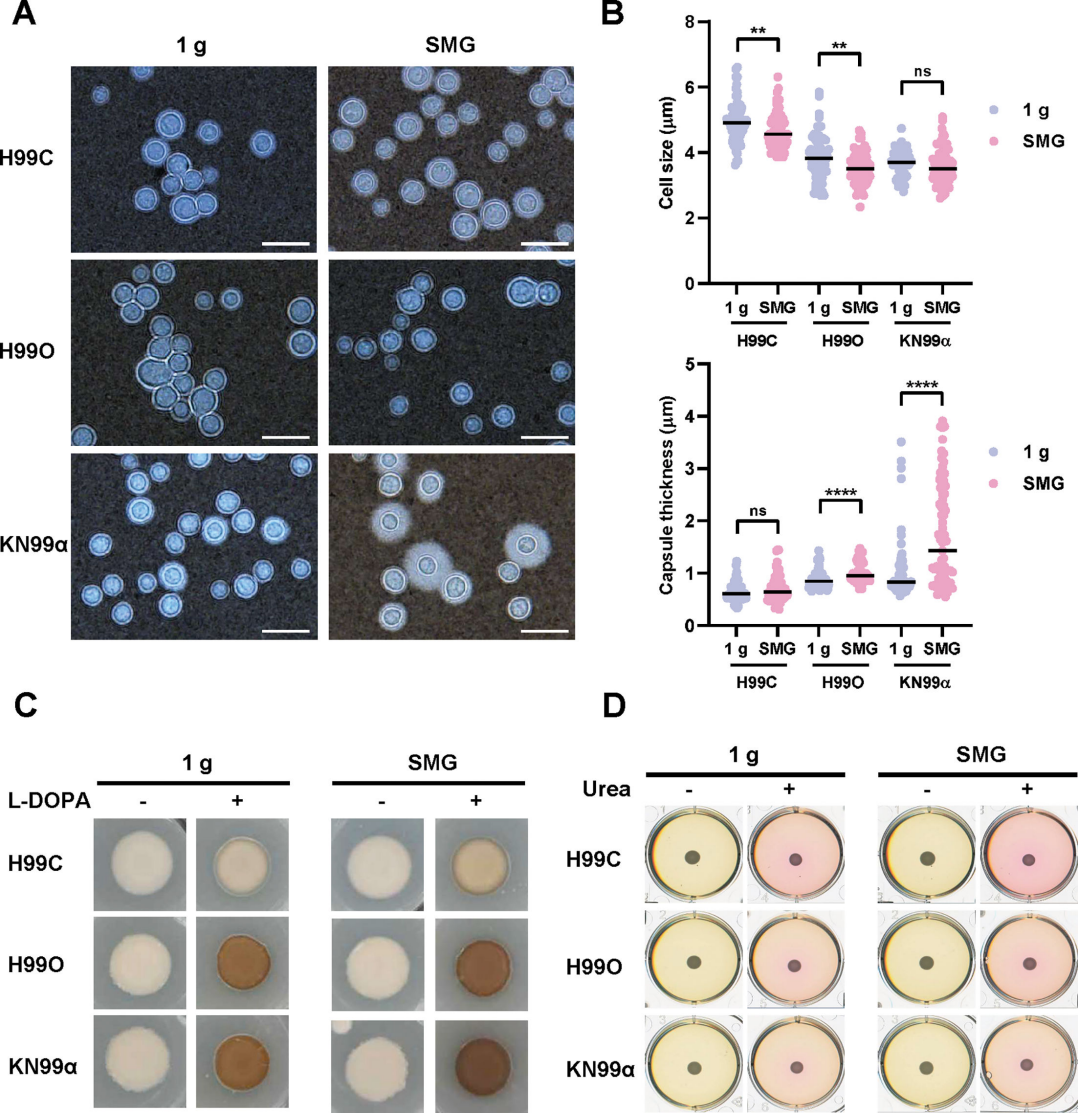
Virulence factors of *C. neoformans* increase under simulated microgravity conditions. Each strain of *C. neoformans* was subjected to capsule induction in MM agar. Capsule formation was evaluated after incubation at 25°C for 3 days. (**A**) Cells and capsule polymorphisms were observed by using India ink staining. Scale bar, 10 µm. 1 g, normal gravity; SMG, simulated microgravity conditions. (**B**) Cell size and capsule thickness were measured and analyzed by ImageJ 1.53t. Statistical analysis was carried out by a two-tailed unpaired *t*-test with a Mann-Whitney test (**, *P* < 0.01; ****, *P* < 0.0001; ns, not significant). (**C**) The melanin production capability of *C. neoformans* was assessed in an MM supplemented with 1 mM L-DOPA, with MM without L-DOPA serving as a control. (**D**) Urease activity of *C. neoformans* was detected in a Christensen’s medium containing 2% wt/vol urea and phenol red as a pH indicator. The well without urea serves as a control. 1 g, normal gravity; SMG, simulated microgravity conditions.

To better understand the effect of SMG on biological features and virulence factors of *C. neoformans*, we initially monitored capsule formation. Capsule of *C. neoformans* is mainly composed of two types of polysaccharides, including glucuronoxylomannan (GXM) and glucuronoxylomannogalactan (GXMGal) (41, 42). It plays an important role in the virulence of this fungal pathogen by inhibiting phagocytosis by macrophages and protecting cells against some stress, such as dehydration and free radicals (43, 44). To induce capsule growth at room temperature, we cultured each strain of *C. neoformans* in minimal media (MM) (45). After 3 days of incubation, we visualized the capsules with India ink staining and measured the cell size and capsule thickness. Our results revealed that the cell sizes were significantly reduced in H99C and H99O, while capsule thicknesses were significantly increased in H99O and KN99α cells growing under SMG conditions compared with the control (Fig. 4A and B). Both cell size reduction and higher capsule formation are considered to increase the virulence of *C. neoformans* (46, 47). Hence, these results suggested that the simulated microgravity potentially promoted the virulence traits of this fungal organism.

Next, we examined the melanin formation of *C. neoformans* under SMG conditions. Melanin is an important virulence attribute that has been reported to be involved in the fungal dissemination from the lung to the brain (48). The melanin production was detected by supplementing MM with l-3,4-dihydroxyphenylalanine (L-DOPA), a precursor of the biological pigment melanin (49), and observing the dark pigment accumulation on the agar compared with the control without L-DOPA. We showed varying melanin production in each fungal strain under normal gravity conditions (50). In addition, the darker colonies compared with the control were observed under SMG (Fig. 4C; Fig. S5A). This strongly indicated that the simulated microgravity conditions promote melanin formation in all tested strains of *C. neoformans*.

Another virulence factor of *C. neoformans* we have tested is the capability to secrete urease, an essential enzyme that promotes the sequestration of cryptococcal cells at microcapillary vessels and subsequently enhances brain invasion (40, 51). We investigated the urease activity using Christensen’s medium with 2% wt/vol urea and phenol red as a pH indicator. Urease will hydrolyze urea to become sodium hydroxide (NaOH), which alters the pH of the medium and results in a change of phenol red’s color from yellow to pink. The results showed that the more pinkish-color agar was found under SMG conditions, which is particularly evident in H99C (Fig. 4D; Fig. S5B).

In summary, we found that SMG conditions increase several disease-associated characteristics, including capsule polymorphisms, melanin formation, and urease activity of *C. neoformans*. These results highlight the impact of SMG on the pathogenic factors of this fungal organism, which could potentially change the pathogenesis of *C. neoformans* in space habitats.

### Simulated microgravity promotes the pathogenicity of *C. neoformans* in the *C. elegans* model

We next asked whether the increasing virulence characteristics in *C. neoformans* impact pathogenicity during cryptococcal infection. To explore this, we utilized the well-established nematode *C. elegans* as a model to study *C. neoformans* pathogenesis. This model organism was chosen because yeast, including cryptococcal species, can serve as the sole food source for *C. elegans* (52, 53), allowing us to observe the pathogenic mechanisms of yeast following ingestion by the worm. The transparent nature of *C. neoformans* colonies also facilitates monitoring of *C. elegans* survival using a dissecting microscope. Additionally, *C. elegans* is typically maintained on solid media, such as nematode growth medium (NGM) agar, making it feasible for incubation in the rotating environment of a clinostat machine during simulated microgravity experiments.

**Fig 5:**
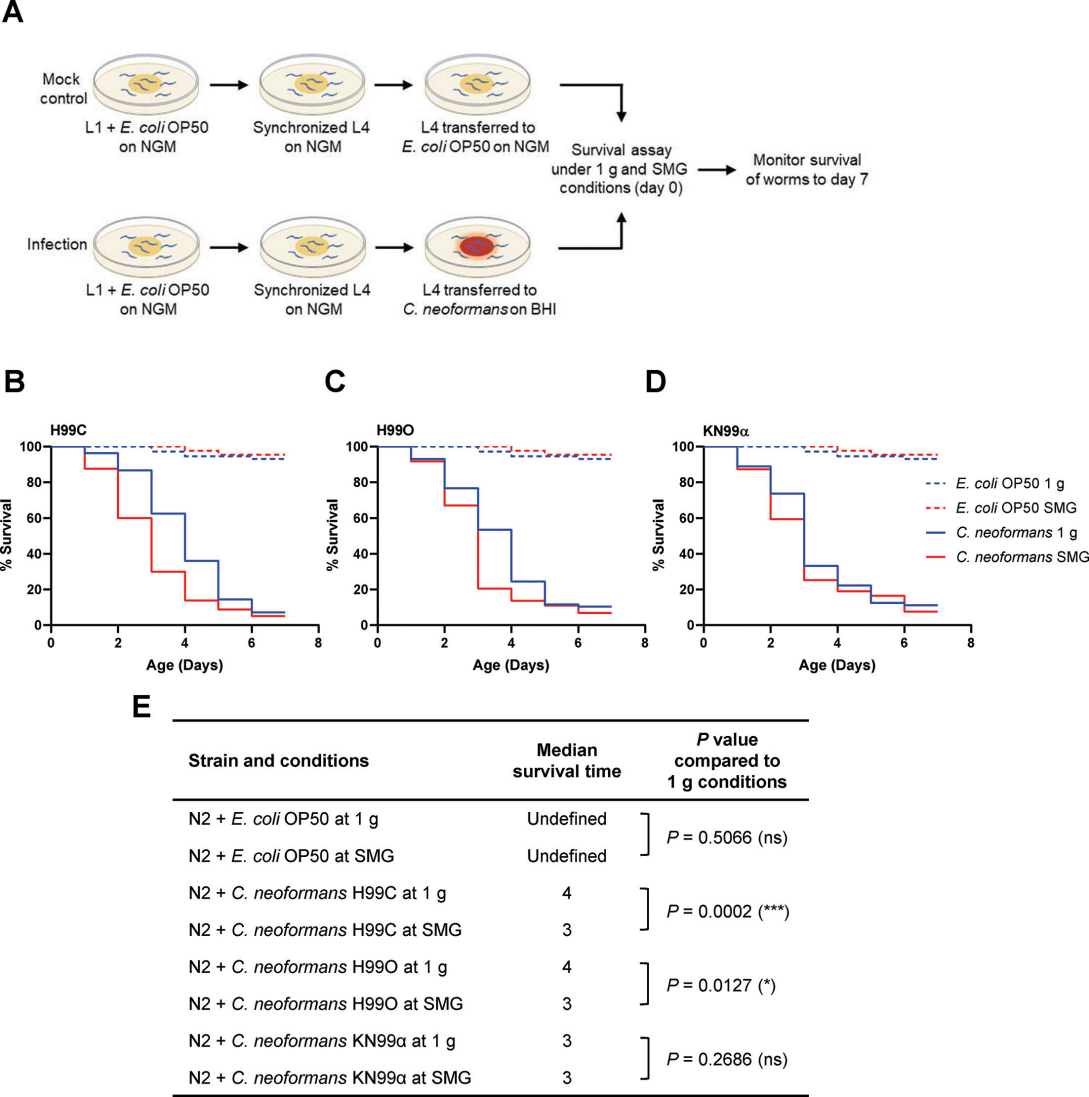
Simulated microgravity enhances killing of *C. elegans* infected with *C. neoformans*. (**A**) A schematic diagram of the *C. elegans-Cryptococcus* killing model. Wild-type *C. elegans* N2 were fed with *E. coli* OP50 on NGM plates from the first larval stage (L1) until they reached the young-adult stage (L4). At this stage, worms were divided into four groups: control-fed *E. coli* OP50 under normal gravity, control-fed *E. coli* OP50 under simulated microgravity, infection with *C. neoformans* under normal gravity, and infection with *C. neoformans* under simulated microgravity. All conditions were maintained at 22°C from day 0 to day 7. Survival percentages of *C. elegans* infected with *C. neoformans* strains H99C (**B**), H99O (**C**), and KN99α (**D**) were assessed. The survival curves were analyzed by Kaplan–Meier analysis, and *P* values were obtained from the log‐rank test (*, *P* < 0.1; ***, *P* < 0.001; ns, not significant). (**E**) Statistical details of survival curves in *C. elegans* for each *C. neoformans* strain are summarized. 1 g, normal gravity; SMG, simulated microgravity conditions.

The young adult (L4) stage worms were transferred to the brain heart infusion (BHI) media containing each strain of *C. neoformans*. The survival curve was represented as days the worms survived (Fig. 5A). Under normal gravity conditions, we demonstrated that fungal infection significantly shortened the survival rates of *C. elegans* compared with the worms fed with *Escherichia coli* OP50 serving as a control (*P* < 0.0001) (Fig. S6). *C. neoformans* strain H99C and H99O appeared to be similarly virulent, while the highest virulent strain is KN99α (Fig. S6), similar to what has been reported previously in the insect *Galleria mellonella* model (50).

Interestingly, our investigation revealed that nematodes infected with *C. neoformans* strains H99C and H99O experienced a significantly reduced survival rate under SMG conditions. Specifically, nematodes infected with H99C showed a markedly reduced survival ratio (Fig. 5B, *P* = 0.0002 and 5E), while those infected with H99O also had a reduced survival fraction, though not as pronounced (Fig. 5C, *P* = 0.0127 and 5E). In contrast, nematodes fed with strain KN99α exhibited no significant change in survival rate under the same conditions (Fig. 5D, *P* = 0.2686 and 5E). These results suggest that simulated microgravity conditions may enhance specific virulence traits in *C. neoformans,* as shown in previous results, and contribute to increased pathogenicity in the *C. elegans* model.

## DISCUSSION

Fungal persistence and adaptation in space habitats remain an underappreciated biosafety concern. Environmental surveys of spacecraft have repeatedly identified medically relevant fungi, including *C. neoformans*, on air and surface samples (20), and microgravity is known to alter host immunity (7–16). Against this backdrop, our findings indicate that simulated microgravity (SMG) conditions can remodel *C. neoformans* biology toward enhanced pathogenic potential. Using a ground-based 3D clinostat that achieves the lowest attainable analog of microgravity of 0.01 g (Fig. 1) (54, 55), we observed a coherent shift across stress tolerance (Fig. 2), antifungal susceptibility (Fig. 3), virulence traits (Fig. 4), and host killing (Fig. 5) that together outline a plausible, membrane-centric mechanism of adaptation.

A key observation is the possible reconfiguration of plasma-membrane properties under SMG. Phenomenologically, SMG increased tolerance to membrane and osmotic challenges while, paradoxically, heightening susceptibility to amphotericin B (AMB). The simplest mechanistic link is altered sterol biology—increased ergosterol abundance and/or changes in sterol organization—that can simultaneously stiffen or stabilize membranes under ionic/osmotic conditions while simultaneously increasing the accessibility of AMB binding sites (33, 34, 56). This interpretation is consistent with biophysical reports from artificial and cellular systems showing microgravity-associated increases in membrane fluidity and changes in microviscosity and lipid packing (57–61). Membrane remodeling in *C. neoformans* plausibly engages capsule and pigment pathways, both membrane-proximal, offering a mechanistic basis for SMG-induced increases in capsule thickness and melanization that support immune evasion and survival (38–40, 48).

Strain background modulated the phenotypic envelope. In our *C. elegans* model, SMG amplified virulence most clearly in the less-virulent H99C/H99O strains, whereas the more hypervirulent KN99α showed little additional effect. This likely reflects a ceiling effect, where high baseline virulence in KN99α constrains further measurable increases under SMG. Importantly, the SMG-linked traits (i.e., smaller cell size in some backgrounds, thicker capsules, and increased urease activity) may represent distinct mechanisms of pathogenesis: enhanced phagocytic uptake with improved intracellular survival and dissemination for small cells (62, 63), impaired macrophage clearance for thick capsules (64), and facilitated microvascular sequestration/brain entry via urease (40). Taken together, these convergent effects suggest that SMG recalibrates multiple virulence programs in parallel.

Our results also help reconcile divergent literature on lipid/sterol regulation under microgravity or spaceflight. *Bacillus cereus* under microgravity shows increased unsaturated fatty acids that preserve membrane flexibility (65), whereas space-flown *C. albicans* downregulated *ERG* genes linked to polyene resistance (27). Such differences likely reflect organism-specific compensation in lipid/sterol pathways—via flux redistribution, intermediate accumulation, or differential *ERG* regulation—as well as cross-talk with stress signaling. Targeted lipidomics, sterol-intermediate quantitation, and transcriptomic/proteomic profiling in *C. neoformans* under SMG should clarify the underlying regulatory mechanisms.

This study has limitations. First, spot assays provide qualitative resolution and may miss subtle growth-rate shifts; liquid CFU readouts were constrained by the obligate aeration needs of *C. neoformans*. Second, we evaluated only one SMG platform and a single temperature setting; comparing multiple analogs, such as the rotating cell culture system (RCCS), high-aspect ratio vessels (HARVs), and the random positioning machine (RPM), along with varied temperatures and radiation co-exposures, would better approximate spaceflight conditions. Finally, while *C. elegans* offers a tractable, agar-compatible host for SMG studies (52, 53, 66), mammalian models will ultimately be required to translate these findings to human risk.

From an operational perspective, our data suggest that microgravity-associated membrane remodeling can reshape antifungal response profiles—most notably by increasing AMB susceptibility while leaving azole and flucytosine MICs largely unchanged. Given the immune alterations observed during spaceflight and documented fungal burden aboard spacecraft, antimicrobial stewardship for long-duration missions should anticipate such shifts, including potential adjustments in first-line therapy or prophylaxis strategies.

In summary, SMG appears to remodel *C. neoformans* membrane biology and linked virulence programs, yielding a phenotype consistent with increased pathogenic potential and, in select genetic backgrounds, higher host mortality. Future work integrating lipidomics/sterol flux analysis, capsule and pigment pathway genetics, and multi-omics with flight-relevant co-stressors will be essential to establish causality and to inform practical countermeasures for astronaut health.

## MATERIALS AND METHODS

### Yeast strains and culture conditions

The strains of *C. neoformans* used in this study, including H99C, H99O, and KN99α, were obtained from BEI Resources, National Institute of Allergy and Infectious Diseases (NIAID). *C. neoformans* was cultured in yeast extract peptone dextrose (YPD) (1% yeast extract, 2% peptone, 2% glucose), MM (15 mM glucose, 10 mM MgSO_4_, 29 mM KH_2_PO_4_, 13 mM glycine, 3 µM thiamine), or RPMI-1640 (Invitrogen) supplemented with 2% glucose, where appropriate and incubated at 30°C (67–69). Yeast growth assays were performed by spotting 10-fold serial dilutions of cells on the indicated solid agar plates before incubating for 2 days.

To assess the effects of stress on *C. neoformans*, stressors including SDS, NaCl, H₂O₂, and NaOH for pH adjustment were added to YPD media. Colony images were analyzed using ImageJ version 1.53t. Colony growth under each condition was quantified by calculating the area under the curve and expressed as a percentage relative to growth on standard YPD agar.

### Simulated microgravity conditions

The simulated microgravity condition is achieved through continuous rotation around two axes by the Gravite 3D clinostat (Space Bio-Laboratories) to rotate samples three-dimensionally (Fig. 1A). Samples were assembled on multi-sample holders, which can mount 25 mL culture flasks, multi-well plates, and petri dishes on each holder’s side. The Gravite 3D clinostat, located at Geo-Informatics and Space Technology Development Agency (GISTDA), Chon Buri, Thailand, was operated according to manufacturer procedures. These conditions created a stable gravity of 0.01 within 10 min after machine operation (70). Cultures that were not rotated (1 g; normal gravity) served as a control for comparison.

### Melanin production and urease activity

Melanin production was measured by incubating the culture in an MM supplemented with 1 mM l-3,4-dihydroxyphenylalanine (L-DOPA; Merck Millipore) (71, 72). Briefly, MM supplemented with L-DOPA was prepared as 2× and filtered sterile. Agar was prepared and autoclaved separately, and after reaching a temperature of 60°C, it was mixed with filtered sterile 2× MM, reaching the final concentration of 1.5% agar. The MM agar without L-DOPA was prepared and used as a control. Then, 3 µL of the fungal inoculum at a turbidity of 3 on the McFarland scale for each tested strain was added to different wells of a 24-well plate containing both control media and media supplemented with L-DOPA, in duplicate. The plates were insulated from light with aluminum foil and incubated at room temperature (25°C) in both simulated microgravity and normal gravity conditions for 3 days.

*C. neoformans* was tested for urease activity by growing on Christensen’s medium (0.1% peptone, 0.5% NaCl, 0.1% glucose, 0.2% KH_2_PO_4_, 0.0012% phenol red) (51, 73). The agar was prepared by adding 2% urea to a pre-autoclaved medium before inoculating with the fungal culture. Control wells without fungal culture were included. Plates were then incubated for 3 days at room temperature under simulated microgravity and normal gravity conditions.

Colony images were analyzed using ImageJ version 1.53t. Pixel intensity values ranging from 0 to 255 gray levels were quantified to assess melanin production. For the urease assay, the pixel intensity distribution of the hue channel, scaled from 0 to 255 and corresponding to 0–360° on the color wheel, was extracted from HSB-transformed images and presented as a histogram.

### Antifungal susceptibility

To determine the antifungal susceptibility, the agar microdilution method was performed as described previously with some modifications (74). Briefly, dilutions of amphotericin B (AMB; Sigma-Aldrich), fluconazole (FLZ; Tokyo Chemical Industry), and 5-fluorocytosine (5FC; Sigma-Aldrich) were prepared from a stock solution in DMSO. Ten microliters of each dilution were dispensed into the individual well of a 24-well plate, followed by the addition of 1 mL of molten YPD and RPMI-1640 agar where appropriate. The final drug concentration in the agar ranged between 0.125 and 2 µg/mL for AMB, 8–128 µg/mL for FLZ, and 0.0625–1 µg/mL for 5FC. Wells containing 1% DMSO were used as a control. The wells were then spotted with 1 µL of the fungal inoculum with a turbidity of 0.5 on the McFarland scale and incubated at room temperature in both simulated microgravity and normal gravity conditions for 2 days. Colony growth was determined by calculating the area under the curve as described above and expressed as a percentage relative to growth on media containing DMSO.

### Staining and microscopy

Staining and microscopy were performed to measure cell and capsule sizes as described earlier (46, 67). Briefly, 5 µL of culture was mixed with the India ink on a glass slide before being subjected to observation using the Upright Fluorescence Microscope BX53 (Olympus Life Science). For each strain, the variation of cell sizes and capsules was determined by looking over 10 random fields of view containing a minimum of 50 regular cells in total at 100 magnification.

### Sterol quantification (ergosterol biosynthesis assay)

Total intracellular sterols were extracted using a method described before with slight modification (68, 75, 76). The cells incubated under normal gravity and simulated microgravity were collected from YPD agar and washed once with sterile distilled water. Samples were collected for total protein quantification. The pellets were then dissolved in 3 mL of 25% alcoholic potassium hydroxide solution and vortexed for 1 min. After mixing, cells were incubated in an 85°C water bath for 1 h. Sterols were extracted by adding 1 mL of sterile distilled water and 3 mL of hexane and vortexing vigorously for 3 min. The hexane layer containing total sterols was transferred to the clean tube stored at −20°C. Prior to analysis, the sterol extract was diluted fivefold in absolute ethanol and spectrophotometrically scanned between 200 and 500 nm with a UV-Vis spectrophotometer (Shimadzu UV-2600). Ergosterol content was calculated as a percentage of the total protein of the cells by the following equations:


$$\%24\left(28\right)DHE=\frac{\left(A_{230}/518\right)\times F}{\text{total protein weight}},$$

$$\%Ergosterol+\%24\left(28\right)DHE=\frac{\left(A_{281.5}/290\right)\times F}{\text{total protein weight}}.$$

### *C. elegans* killing assay

The wild-type *Caenorhabditis elegans* strain, N2 Bristol, obtained from the *Caenorhabditis* Genetics Center, was used in this study. All worms were age‐synchronized using hypochlorite treatment (77). The first stage (L1) larvae were transferred into fresh NGM plates and fed on *E. coli* strain OP50 at 20°C as previously described (66).

For *C. elegans*-cryptococcal infection, yeast cells were inoculated into 3 mL of YPD and grown at 30°C for 48 h. Cultures were adjusted to 1.5 McFarland and spread on 35 mm tissue-culture plates containing BHI agar (Himedia) supplemented with 100 µg/mL ampicillin and 50 mM 5′-fluorodeoxyuridine (FUdR; Merck Millipore). The plates were incubated at 30°C overnight. Then, approximately 40–50 worms at the young adult developmental stage (L4) were transferred from a lawn of *E. coli* OP50 on NGM to a lawn of the yeast to be tested on BHI medium, incubated at 22°C under normal gravity and simulated microgravity conditions as day 0 (78), and examined for viability at 24 h intervals with a dissecting microscope until day 7. Worms were considered dead when they did not respond to touch with a platinum wire or did not show pharyngeal pumping movement. Each experimental condition was tested in duplicate. Nematodes fed with *E. coli* OP50 were used as mock-infected controls.

### Statistical analysis

For statistical analysis, GraphPad Prism 8.4.2 was used. Data from each experiment were checked for normality. After passing the normality test, data were analyzed by a two-tailed unpaired *t*-test with a Mann–Whitney test, and two-way analysis of variance with Sidak’s multiple comparisons test. For the *C. elegans* killing assay, plotting of killing curves and estimation of difference in survival were performed with Kaplan–Meier analysis, and *P* values were calculated using the log-rank test.

## Data Availability

The raw data that support the findings of this study are openly available in Figshare under https://figshare.com/s/0bcece08cbd2effc2dc7.

## SUPPLEMENTAL MATERIAL

Supplemental figures aem.01435-25-s0001.pdf Figures S1 to S6.

ASM does not own the copyrights to Supplemental Material that may be linked to, or accessed through, an article. The authors have granted ASM a non-exclusive, world-wide license to publish the Supplemental Material files. Please contact the corresponding author directly for reuse.
