# Supplementary material for: Effects of simulated microgravity on biological features and virulence of the fungal pathogen Cryptococcus neoformans
Source: Appl Environ Microbiol. 2025 Sep 30;91(10):e01435-25. doi: 10.1128/aem.01435-25 (PMC12542639; doi:10.1128/aem.01435-25)
Supplement: Supplemental figures — Figures S1 to S6. [file aem.01435-25-s0001.pdf]

SUPPLEMENTARY INFORMATION

Effects of simulated microgravity on biological features and virulence of the fungal pathogen *Cryptococcus neoformans*

Tanaporn Phetruen[1], Salinthip Thongdechsri[2], Muthita Khongthongdam[1], Sittiporn Channumsin[3], Krai Meemon[2], and Sittinan Chanarat[1,*]

[1] Laboratory of Medical Molecular Mycology, Department of Biochemistry and Center for Excellence in Protein and Enzyme Technology, Faculty of Science, Mahidol University, Bangkok, Thailand

[2] Department of Anatomy, Faculty of Science, Mahidol University, Bangkok, Thailand

[3] Space Technology Research Centre, Geo-Informatics and Space Technology Development Agency (GISTDA), Chonburi, Thailand

* Email: sittinan.cha@mahidol.ac.th

Supplementary Figure 1

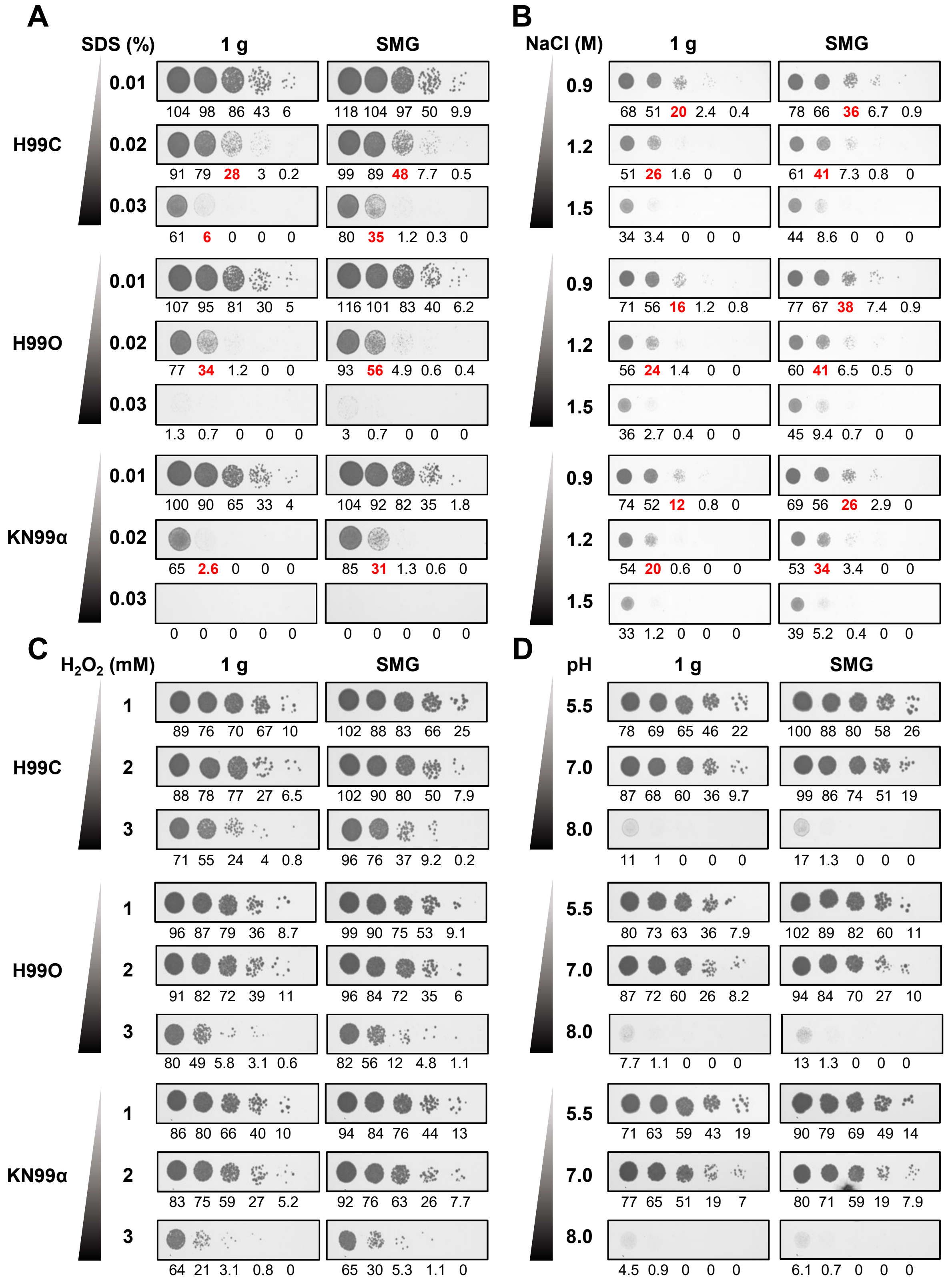

Supplementary Fig. 1: Simulated microgravity marginally affects stress tolerance of *C. neoformans*. Colony images of *C. neoformans* grown on YPD medium supplemented with the indicated stress-inducing agents were analyzed using ImageJ 1.53t: (**A**) SDS (membrane stress), (**B**) NaCl (osmotic stress), (**C**) H_2O_2 (oxidative stress), and (**D**) NaOH-adjusted medium (pH stress). Colony growth was quantified by calculating the area under the curve (AUC) and expressed as a percentage relative to growth on standard YPD agar (Figure 1D, control). Data points showing the marked differences between 1 g and SMG conditions are highlighted in bold red. 1 g; normal gravity, SMG; simulated microgravity conditions.

Supplementary Figure 2

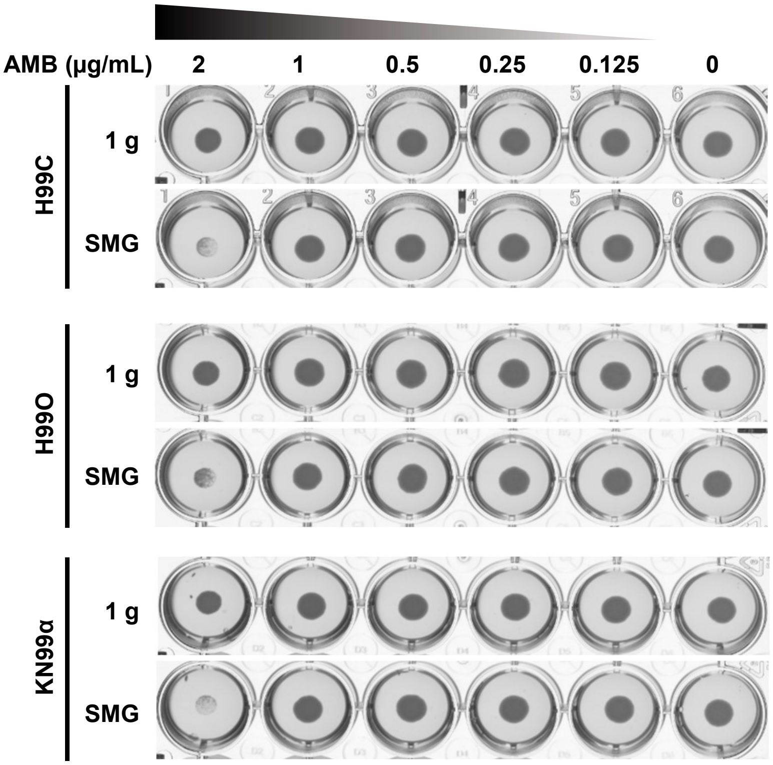

Supplementary Fig. 2: Simulated microgravity condition slightly increases antifungal susceptibility in *C. neoformans*. Each strain was diluted to 0.5 on the McFarland scale and spotted on YPD agar supplemented with the indicated concentration of amphotericin B (AMB). Cells were incubated under simulated microgravity and normal gravity conditions for 2 days at 25°C and photographed. Figures were the representatives of duplicate data. 1 g; normal gravity, SMG; simulated microgravity conditions.

Supplementary Figure 3

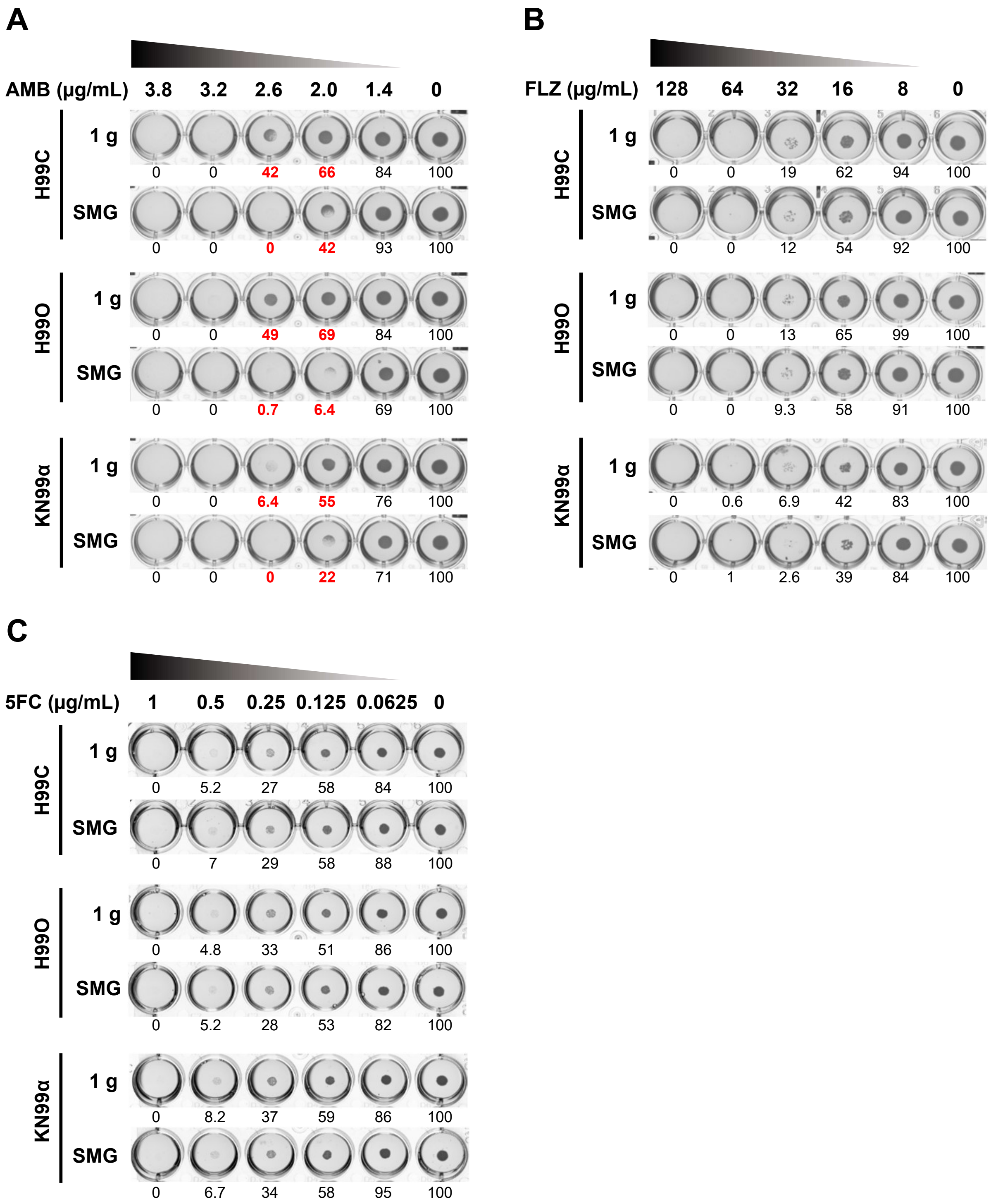

Supplementary Fig. 3: Antifungal susceptibility in *C. neoformans* is minimally enhanced under simulated microgravity conditions. Colony images of *C. neoformans* grown on medium supplemented with the indicated concentration of antifungal agents were analyzed using ImageJ 1.53t: (**A**) amphotericin B (AMB), (**B**) fluconazole (FLZ), and (**C**) 5-fluorocytosine (5FC). Colony growth was quantified by calculating the area under the curve (AUC) and expressed as a percentage relative to growth on medium supplemented with DMSO. Data points showing the marked differences between 1 g and SMG conditions are highlighted in bold red. 1 g; normal gravity, SMG; simulated microgravity conditions.

Supplementary Figure 4

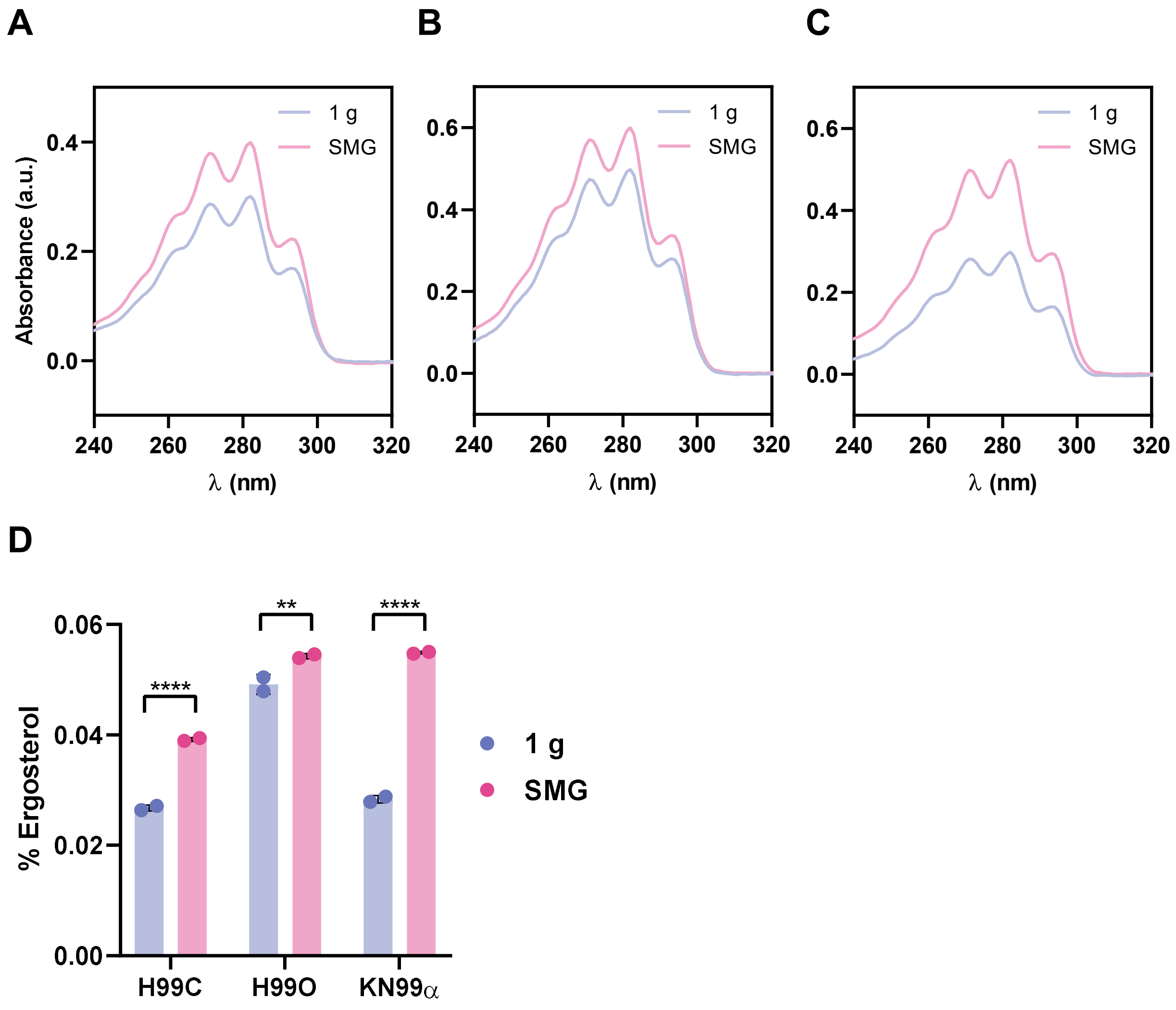

Supplementary Fig. 4: Simulated microgravity condition affects ergosterol in the fungal cell membrane. The UV absorption spectra of ergosterol extracted from *C. neoformans* strains H99C **(A)**, H99O **(B)**, and KN99α **(C)** incubated under normal gravity (1 g; purple) and simulated microgravity (SMG; pink) were compared. **D** Percentages of ergosterol per total protein weight were calculated and analyzed. Statistical analysis was carried out by two-way ANOVA with Sidak's multiple comparisons test (**, $P < 0.01$; ****, $P < 0.0001$).

Supplementary Figure 5

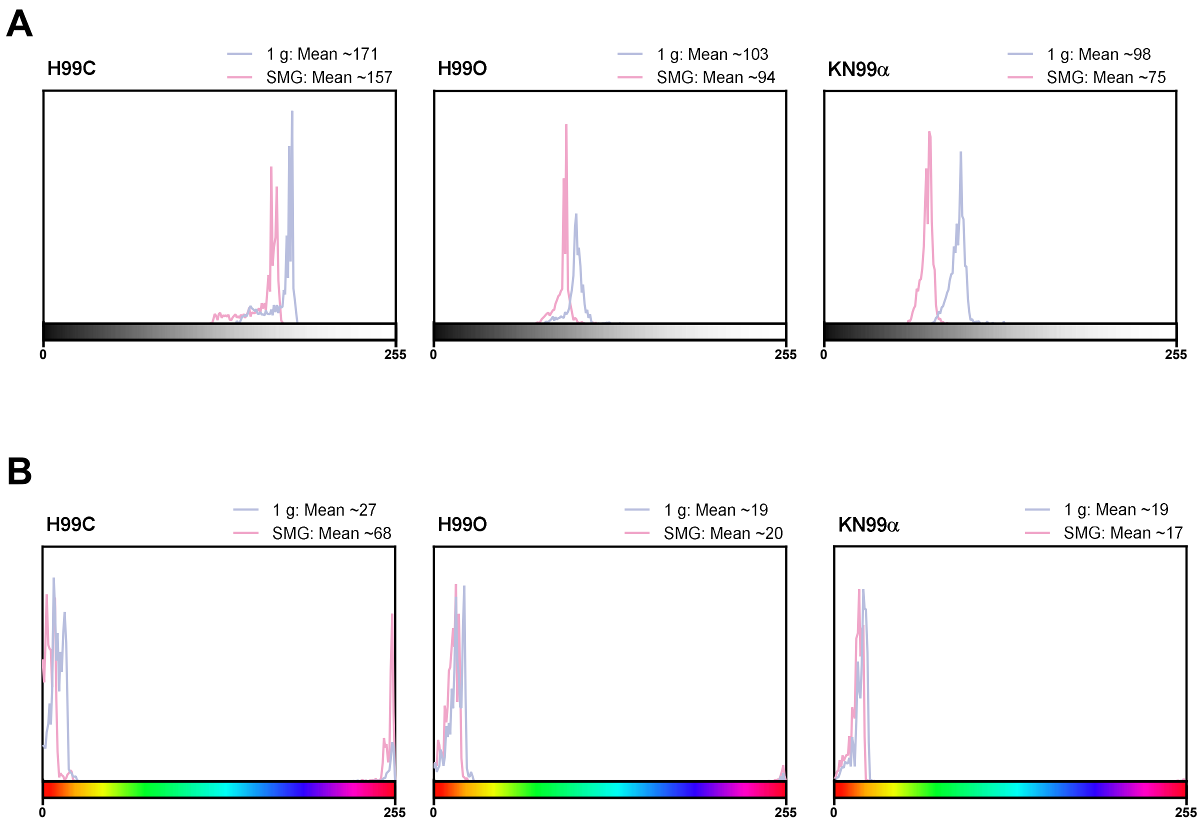

Supplementary Fig. 5: Simulated microgravity caused a shift in the histograms of both melanin production and urease secretion in *C. neoformans*. Colony images of *C. neoformans* were analyzed using ImageJ 1.53t. For melanin (corresponding to Fig. 4C), pixel intensity values (0–255 gray levels) were quantified, with the x-axis of the histogram representing pixel intensity and the y-axis representing pixel frequency (**A**). For urease activity (corresponding to Fig. 4D), pixel intensity distribution of the hue channel extracted from HSB-transformed images were similarly quantified and displayed as histograms (**B**). Hue values on the x-axis (0–255, mapped to 0–360°) correspond to angles on the color wheel, representing dominant color components within the images, while the y-axis indicates pixel frequency. 1 g; normal gravity, SMG; simulated microgravity conditions.

Supplementary Figure 6

A

B

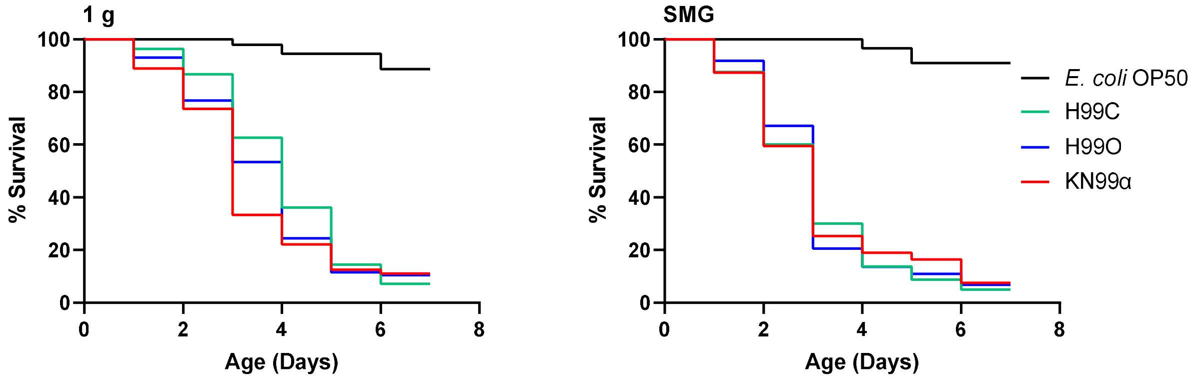

C

Strain and conditions	Median survival time	*P* value compared to *E. coli* OP50	*P* value compared to other strain
N2 + *E. coli* OP50 at 1 g	Undefined	-	-
N2 + *C. neoformans* H99C at 1 g	4	****	ns
N2 + *C. neoformans* H99O at 1 g	4	****	ns
N2 + *C. neoformans* KN99α at 1 g	3	****	ns
N2 + *E. coli* OP50 at SMG	Undefined	-	-
N2 + *C. neoformans* H99C at SMG	3	****	ns
N2 + *C. neoformans* H99O at SMG	3	****	ns
N2 + *C. neoformans* KN99α at SMG	3	****	ns

Supplementary Fig. 6: Survival curves of *C. elegans* infected with different strains of *C. neoformans* were analyzed. Wild-type *C. elegans* N2 at the young-adult (L4) stage were infected with *C. neoformans* strains H99C (green), H99O (blue), and KN99α (red). Plates were incubated at 22°C under normal gravity (1 g; **A**) and simulated microgravity (SMG; **B**) until day 7. Worms fed with *E. coli* OP50 (black) were served as mock-infected controls. The median lethal time (LT50) was analyzed by simple logistic regression and *P*-values of survival curves were obtained from the log-rank test (****, $P < 0.0001$; ns, not significant). **C** The statistical details for each condition are summarized.
